# Editorial: Mitral valve disease: in memoriam Adrian Chester

**DOI:** 10.3389/fcvm.2026.1954760

**Published:** 2026-08-19

**Authors:** Michel Pucéat, Joy Lincoln, Stéphane Zaffran

**Affiliations:** 1 Aix-Marseille Université, INSERM U1260, INRAE U1263, C2VN, Marseille, France; 2 Department of Pediatrics, Division of Pediatric Cardiology, Medical College of Wisconsin, Milwaukee, WI, United States; 3 Herma Heart Institute, Children’s Wisconsin, Milwaukee, WI, United States; 4 Aix-Marseille Université, INSERM, MMG U1251, Marseille, France

**Keywords:** congenital heart defect, genetics, mechanobiology, mitral valve, physiopathology, transcriptomics

Mitral valve (MV) diseases encompass a broad spectrum of disorders, ranging from congenital malformations and mitral valve prolapse to progressive myxomatous degeneration, annular dilatation, and calcification. Although these conditions differ in their clinical presentation and natural history, they share common complexities. The mitral valve is a highly dynamic tissue for which the development, homeostasis, and function involve complex interaction between multiple cell types, extracellular matrix components, mechanical forces, and environmental cues. Over the past decades, major advances have transformed our understanding of mitral valve biology. Genetic studies have identified several genes associated with mitral valve prolapse and inherited valve disorders, while advances in developmental biology, transcriptomic approaches, mechanobiology, and cardiovascular imaging have revealed an increasing complex picture of valve formation and disease. However, despite these improvements, the genetic and molecular mechanisms underlying most mitral valve diseases remains poorly understood. The emergence of mitral valve disease at different stages of life further suggests that developmental susceptibility may interact with acquired factors, including aging, inflammation, metabolic alterations, mechanical stress, and environmental influences to progressively alter valve homeostasis.

This Research Topic, “*Mitral Valve Disease*” has been dedicated to the memory of Adrian Chester. This issue brings together contributions addressing the basic biology and the pathology of the mitral valve from embryonic development to adult diseases. The Research Topic reflects the multidisciplinary nature of the field, encompassing genetic, development, cellular and molecular biology, artificial intelligence, biomarkers, and therapy.

Dr. Adrian H. Chester was a brilliant scientist and an incredibly positive man. Born with a complex cardiac congenital disease, he obtained a bachelor's in science in Pharmacology from Portsmouth Polytechnic (UK) in 1986. After a period working in drug discovery in a pharmaceutic company, he moved to the laboratory of Professor Sir Magdi Yacoub at the Harefield Hospital Heart Science for his PhD in 1989. Indeed, Professor Sir Magdi Yacoub had saved his life by performing a Fontan surgery when he was a young 9-year-old child. He worked for a few years on cardiac vascular biology. In 2002, he was promoted to Senior Lecturer in 2002 within the National Heart and Lung Institute. Then, he dedicated his research to the role of valve endothelial and interstitial cells in the regulation of heart valve function, and the pathogenesis of valve diseases. He was an inspiring partner in the Transatlantic MITRAL network of excellence funded by the Leducq Foundation (2007–2013) focused on mitral valve disease. With more than 160 scientific publications, Adrian made major contributions to valve biology, cardiovascular disease, and bioengineering. Yet his legacy extends beyond his impressive publication record. He inspired colleagues and younger scientists through his scientific rigor, creativity, openness, and enthusiasm.

Despite substantial progress in surgical repair and replacement, including minimally invasive and transcatheter interventions, effective strategies to prevent or modify the biological processes driving mitral valve disease remain poorly understood. The future of the field will require a deeper understanding of how genetic predisposition, developmental programs, cellular interactions, extracellular matrix remodeling, and mechanical forces converge to determine disease susceptibility and progression. Advances in high-resolution imaging, single-cell and spatial technologies, artificial intelligence, and bioengineering offer unprecedented opportunities to address these challenges and to develop more personalized medicine to patient care.

We hope that this dialogue between basic, translational, and clinical research will contribute to a better understanding of mitral valve disease, as well as to the development of more precise strategies for diagnosis, risk stratification, prevention, and treatment.

